# Long non-coding RNAs in response to Ebola virus vaccine-induced immunity

**DOI:** 10.3389/fimmu.2025.1695514

**Published:** 2026-02-10

**Authors:** Izabela Mamede, Thomaz Luschër-Dias, Isabelle Franco Moscardini, Patrícia Gonzales-Dias, Bárbara Marinho, Fernando Marcon, Thiago Dominguez Crespo Hirata, Michael Eichberg, Donata Medaglini, Ali M. Harandi, Claire-Anne Siegrist, Tom H. M. Ottenhoff, Francesco Santoro, Marylyn M. Addo, André Gonçalves, Daniela M. Ferreira, Rafael Polidoro, Glória R. Franco, Paulo P. Amaral, Helder Nakaya

**Affiliations:** 1 Department of Biochemistry and Immunology, Institute of Biological Sciences, Universidade Federal de Minas Gerais, Belo Horizonte, Brazil; 2 Department of Clinical and Toxicological Analyses, School of Pharmaceutical Sciences, Universidade de São Paulo, São Paulo, Brazil; 3 Bios-Therapy, Physiological Systems for Health S.p.A., Sansepolcro, Italy; 4 Oxford Vaccine Group, Department of Paediatrics, University of Oxford, Oxford, United Kingdom; 5 Laboratory of Genetics and Molecular Cardiology (LGCM), InCor/Clinical Hospital FMUSP (HCMUSP), São Paulo, Brazil; 6 Centre of Omics Technologies (CTO), School of Pharmaceutical Sciences, University of São Paulo, São Paulo, Brazil; 7 Strategic Alliances at Merck Research Laboratories, Boston, MA, United States; 8 Department of Medical Biotechnologies, Laboratory of Molecular Microbiology and Biotechnology, University of Siena, Siena, Italy; 9 Department of Microbiology & Immunology, Institute of Biomedicine, Sahlgrenska Academy, University of Gothenburg, Gothenburg, Sweden; 10 Vaccine Evaluation Center, BC Children’s Hospital Research Institute, University of British Columbia, Vancouver, BC, Canada; 11 Center of Vaccinology, Department of Pathology and Immunology, Faculty of Medicine, University of Geneva, Geneva, Switzerland; 12 Leiden University Center of Infectious Diseases (LUCID), Leiden, Netherlands; 13 Dipartimento di Biotecnologie Mediche, Università di Siena, Siena, Italy; 14 Department of Pediatrics, Indiana University School of Medicine, Indianapolis, IN, United States; 15 INSPER Institute of Education and Research, São Paulo, Brazil; 16 Hospital Israelita Albert Einstein, São Paulo, Brazil; 17 Institut Pasteur de São Paulo, São Paulo, Brazil; 18 Institute for Infection Research and Vaccine Development (IIRVD), University Medical Centre Hamburg-Eppendorf, Hamburg, Germany; 19 Department for Clinical Immunology of Infectious Diseases, Bernhard Nocht Institute for Tropical Medicine, Hamburg, Germany; 20 German Centre for Infection Research, partner site Hamburg-Lübeck-Borstel-Riems, Hamburg, Germany

**Keywords:** Ebola (EBOV), lncRNA, RNA, systems biology, vaccine

## Abstract

Long noncoding RNAs (lncRNAs) have emerged as critical regulators of gene expression, yet their role in shaping human responses to vaccination remains largely uncharacterized. Here, we analyzed RNA-sequencing data from three independent human cohorts vaccinated with the rVSVΔG-ZEBOV-GP Ebola vaccine to profile lncRNA expression dynamics. Using differential expression analysis and correlation meta-analysis across cohorts, we identified an expression signature with several lncRNAs, including *LEF1-AS1* and *DOCK8-AS1*, that exhibit conserved transcriptional activation following vaccination. Correlation of lncRNA expression with gene targets and IgG titers revealed putative roles for lncRNAs in regulating and/or participate in both innate immune responses and adaptive antibody production. Functional enrichment of lncRNA co-expressed protein-coding genes highlighted involvement in T-cell differentiation, interferon signaling, and leukocyte activation. Integrating global run-on sequencing data and comparative transcriptomic analysis across other vaccine studies suggests that *LEF1-AS1* modulation is distinctively associated with Ebola vaccination. Our findings demonstrate that lncRNAs are potential integral components of the human vaccine response and provide a foundation for future mechanistic studies targeting noncoding RNA regulation of immunity.

## Graphical Abstract

**Figure:**
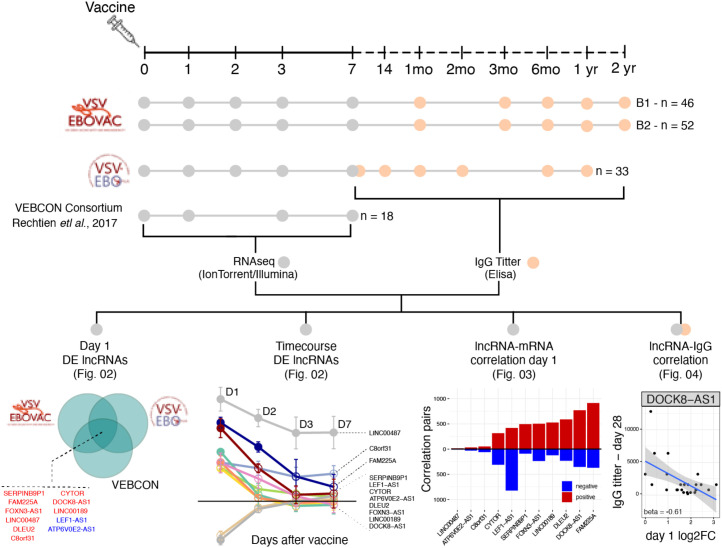

## Introduction

Ebola virus disease (EVD) caused by *Orthoebolavirus zairense* is a severe hemorrhagic fever with a fatality rate ranging from 50% to 90%. The disease is highly contagious, and from 2014 to 2016, an Ebola outbreak in West Africa killed over 11,000 people, while between 2018 and 2020, the outbreak in the Democratic Republic of Congo (DRC) resulted in 3,323 cases and 2,299 deaths. In the aftermath of these outbreaks, coordinated efforts between research consortia, governments, global public health entities, and the private sector resulted in the approval of two treatments by the U.S. Food and Drug Administration (FDA) for EVD caused by *O. zairense*; Inmazeb^®^, a combination of three monoclonal antibodies, and Ebanga^®^, a single monoclonal antibody (Food and Drug Administration [FDA], 2024); and the development of an efficacious and safe vaccine, VSVΔG-ZEBOV-GP. This recombinant Vesicular Stomatitis Virus (VSV) expresses the Zaire Ebola virus (ZEBOV) glycoprotein (GP), and its efficacy was evaluated during the 2014–2016 outbreak in Guinea. rVSV-ZEBOV is the first Ebola vaccine that has been pre-approved for use in response to Ebola outbreaks and has recently been used, showing 84% effectiveness (1). In addition to the single-dose rVSVΔG-ZEBOV-GP vaccine (ERVEBO^®^), a two-dose heterologous regimen using Ad26.ZEBOV and MVA-BN-Filo (Zabdeno^®^/Mvabea^®^) have been licensed for prophylactic use and have been shown to be safe and immunogenic in adults and children in Africa and elsewhere (2, 3).

Systems vaccinology literature demonstrates the relevance of high-throughput techniques correlating gene expression before and after vaccination with the higher quality and persistence of antibodies, guiding improvements in vaccine design and delivery (4–6). The VSV-EBOVAC and VSV EBOPLUS consortia sought to identify important correlates of protection during vaccination using VSVΔG-ZEBOV-GP in adult cohorts in Europe, Africa, and North America using transcriptomics. They analyzed peripheral blood changes induced by the VSVΔG-ZEBOV-GP vaccine and revealed that the peak perturbation occurred one day after vaccination administration of the vaccine (6, 7). The study showed early immune response activation with increased type I and II interferon response-related genes and myeloid cell-associated markers expression. In contrast, genes of circulating effector cells, such as cytotoxicity-associated genes, are downregulated in the peripheral blood cells of vaccinated individuals (4–6). However, these analyses have primarily focused on protein-coding genes, leaving the role of non-coding RNA largely unexplored.

LncRNAs are long (>200 nucleotides) non-coding regulatory RNAs that can subtly regulate gene expression through RNA–RNA, RNA–DNA, and RNA–protein interactions (8). Our group and others have previously demonstrated the relevance of lncRNAs in immune modulation, including cytokine production (9), myeloid cell activation (10), and T and B cell differentiation (11). In addition to these general roles, specific lncRNAs have been functionally linked to key immune mediators. For example, THRIL modulates TNFα expression (12), a cytokine that orchestrates early antiviral and inflammatory responses and can therefore influence both the control of viral infections and the strength of vaccine-induced immune responses. Other lncRNAs have been more directly connected to viral pathogenesis: BISPR is an interferon-stimulated gene (ISG) that is upregulated upon Hepatitis C Virus (HCV) infection (13), NF-κB-induced EGOT dampens the interferon response and promotes HCV replication (14), and, more recently, Katz et al. used machine learning in a patient cohort to identify circulating lncRNAs that can serve as predictors of severe dengue (15). Several other lncRNAs have also been associated with immune responses to viral infections, such as influenza, dengue, and yellow fever, and some are even being investigated as therapeutic targets in cancer and inflammatory diseases (16).

Despite its potential to generate potent immunological memory (11, 17), the involvement of lncRNAs in the immune response to the Ebola vaccine has not been characterized. Investigating the changes in lncRNAs in the vaccinated cohorts may reveal additional immune response mechanisms associated with improved outcomes following VSVΔG-ZEBOV-GP vaccination. Here, we analyzed early lncRNA response upon Ebola vaccination and correlated their expression with IgG titer quantification obtained from three independent cohorts of volunteers vaccinated with VSVΔG-ZEBOV-GP. The goal was to detect differentially expressed (DE) lncRNAs that could mediate the long-term humoral protection conferred by the vaccine. In all cohorts, we detected 11 lncRNAs that were consistently differentially expressed on day 1 following vaccination. Our analysis revealed three lncRNAs—*FAM225A*, *DOCK8-AS1*, and *LEF1-AS1*—that were robustly correlated with IgG titers at several time points after vaccination. Moreover, pairing affected pathways with differentially expressed lncRNAs suggested a potential regulatory role over their associated genes, which are involved in both innate and adaptive immune responses.

## Results

### rVS-ZEBOV vaccination induces consistent day 1 lncRNA expression across cohorts

**Figure 1:**
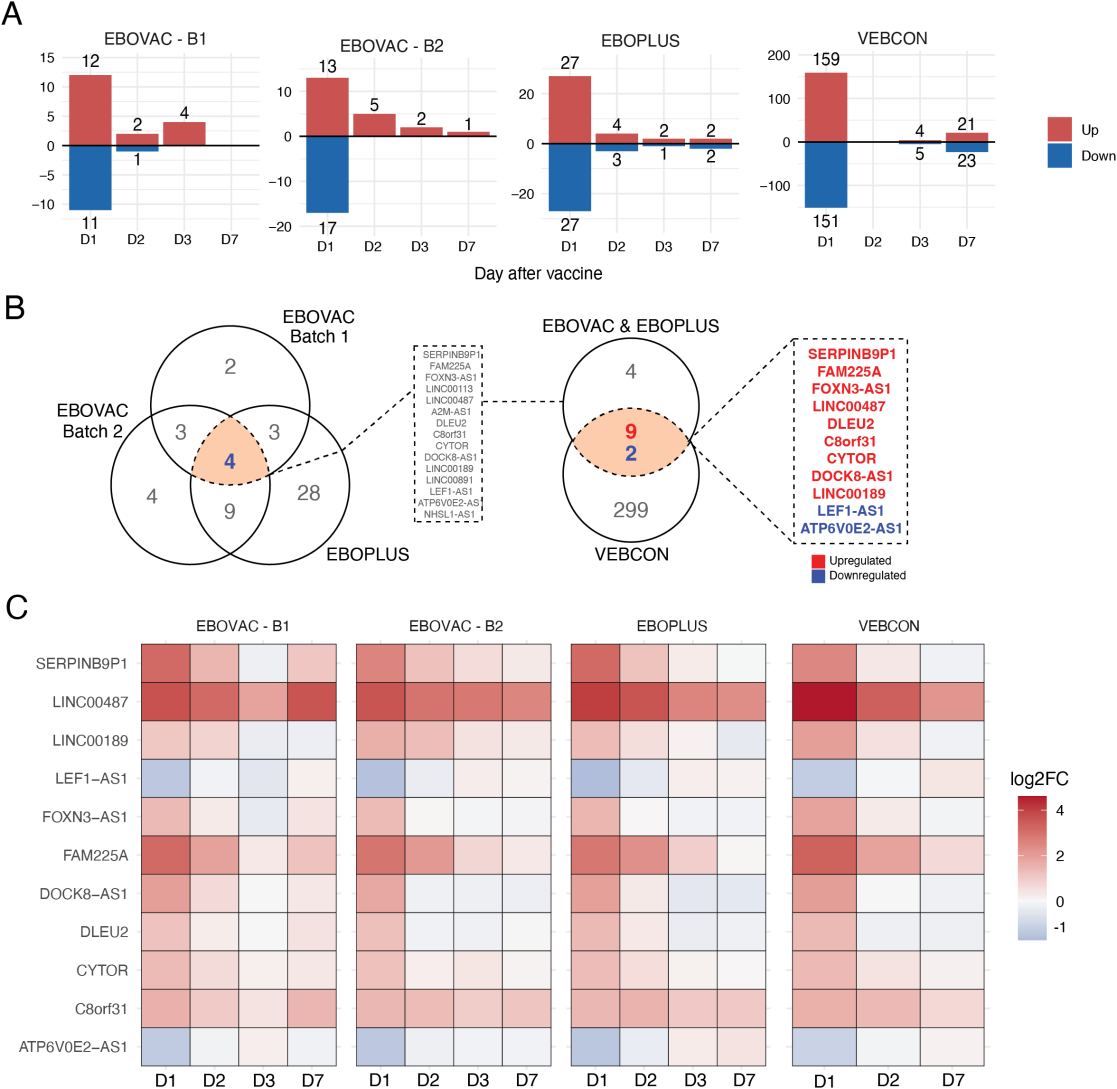
Selected long non-coding RNA (lncRNA) expression is consistent across time points and cohorts following rVSVΔG-ZEBOV-GP vaccination. **(A)** Number of differentially expressed lncRNAs on days 1, 2, 3, and 7 after vaccination in the three different cohorts. **(B)** Venn diagram representation of 11 consistently differentially expressed lncRNAs in all three vaccine cohorts on day 1 after vaccination. Blue: downregulated lncRNAs; red: upregulated lncRNAs. **(C)** Heatmap analysis showing the time course of the log_2_fold change of the 11 common day 1 differentially expressed (DE) lncRNAs shared between all cohorts.

We integrated three distinct cohorts of vaccinated volunteers in our study: VSV EBOVAC (Geneva, n = 98, two batches), VSV EBOPLUS (USA, n = 33), and VEBCON (Germany, n = 18) (**Figure 1**). Volunteers from all cohorts received one dose of the rVSV-ZEBOV vaccine on day zero and had their blood collected just before receiving the vaccine (day 0) and on subsequent days (1, 2, 3, and 7) for RNA sequencing analysis. Volunteers returned at several time points during the 2 years following the vaccination to provide blood for IgG titer quantification using ELISA. RNAseq and IgG titer results were then analyzed to obtain the consistently differentially expressed lncRNAs on day 1 and time course differential expression analysis of those lncRNAs, meta-analysis of the correlation between lncRNA and mRNA expression, and meta-analysis of the correlation between lncRNA expression and IgG titers.

In all cohorts, the peak of gene expression perturbation following vaccination occurred on day 1 (**Figure 1A**). On this day, the rVSV–ZEBOV vaccine elicited 12 upregulated and 11 downregulated lncRNAs in batch 1 EBOVAC volunteers, 13 upregulated and 17 downregulated lncRNAs in batch 2 EBOVAC volunteers, 27 upregulated and 27 downregulated lncRNAs in EBOPLUS volunteers, and 159 upregulated and 151 downregulated lncRNAs in VEBCON volunteers (**Figure 1A**). The number of differentially expressed lncRNAs in the VEBCON cohort was consistently higher than that in the EBOVAC and EBOPLUS cohorts, which can be attributed to the sequencing technology used for that cohort (Illumina) compared to that used in the other cohorts (IonTorrent). As the vaccine transcriptomic perturbation was consistently higher on day 1 than on days 2, 3, and 7 after the vaccine in all cohorts (**Figure 1A**), we decided to detect the lncRNAs that were consistently DE in all groups of volunteers on the first day post-vaccination (**Figure 1B**). Altogether, 15 lncRNAs (11 upregulated and four downregulated) were differentially expressed in both the EBOVAC and EBOPLUS (batch 1 and batch 2) cohorts, 11 of which were also differentially expressed in the VEBCON cohort (**Figure 1B**; **Supplementary Figure S1A**). From these 11 consensus DE lncRNAs between the three cohorts, nine were upregulated (*SERPINB9P1*, *FAM225A*, *FOXN3-AS1*, *LINC00487*, *DLEU2*, *C8orf31*, *CYTOR*, *DOCK8-AS1*, and *LINC00189*) and two were downregulated (*LEF1-AS1* and *ATP6V0E2-AS1*) (**Figure 1B**). The common lncRNAs that were mostly upregulated on day 1 were *LINC00487* and *FAM225A*, and the most downregulated was *LEF1-AS1* (**Figure 1C**). Most of the shared vaccine-induced lncRNAs returned to baseline levels on days 2, 3, and 7, except for *LINC00487*, which remained upregulated in all cohorts until day 7 (**Figure 1C**). In the three groups of volunteers analyzed on day 2, *FAM225A* was upregulated, while this lncRNA was still upregulated on day 3 in the VEBCON patient cohort (**Figure 1C**; **Supplementary Figure S1B**), but it returned to baseline on day 7 post-vaccination.

### Vaccine-induced lncRNAs show correlated expression with genes of key immune pathways and cells

lncRNAs can directly regulate the expression of protein-coding genes through lncRNA–RNA, lncRNA–DNA, and lncRNA–protein interactions (8). This regulation is particularly relevant for other immune-related phenotypes, such as viral and bacterial infections, where specific lncRNAs interact with viral components or immune regulators to modulate interferon responses, inflammation, antigen presentation, and even viral replication. To explore whether consensus day 1 VSV-ZEBOV-affected lncRNAs could influence the expression of other immune-related genes, we performed a correlation analysis between the expression of the 11 lncRNAs at day 1 and the expression of all other genes in the genome in each cohort and integrated those results using a meta-analysis approach.

**Figure 2:**
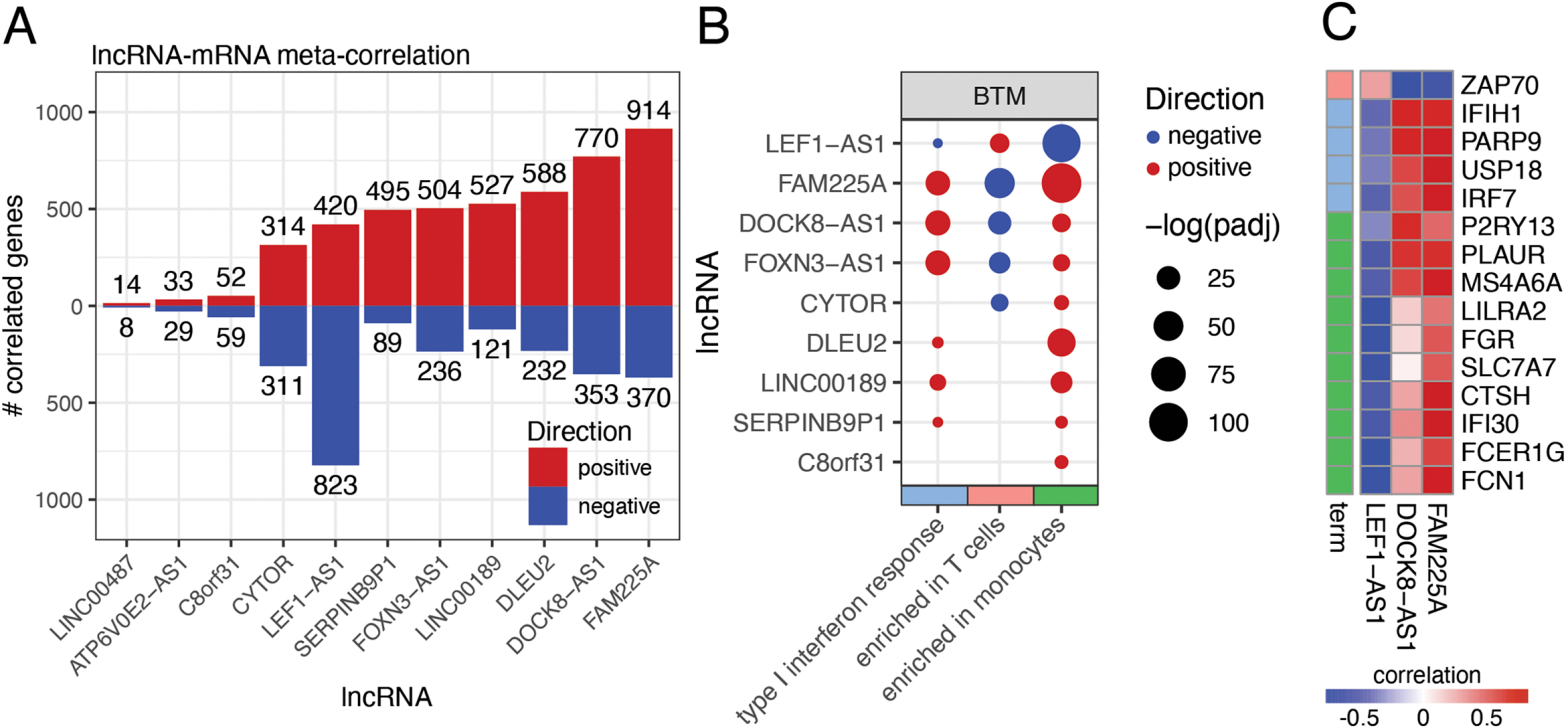
Day 1 vaccine-induced lncRNAs have correlated expression with key immune genes, pathways, and cells across cohorts. **(A)** Number of meta-analysis significant correlation pairs between day 1 vaccine-induced lncRNAs and messenger RNA (mRNAs). Blue: negative correlation pairs; red: positive correlation pairs. **(B)** Overrepresentation analysis of blood transcriptional modules enriched among mRNAs positively (red) and negatively (blue) correlated with vaccine-induced lncRNAs. They are separated according to their BTM (Blood Transcription Module). **(C)** Top positive (red) and negative (blue) correlated mRNAs with lncRNAs FAM225A, LEF1-AS1, and DOCK8-AS1 and the GO terms (term) they are contained in.

The lncRNAs that showed the highest number of meta-correlated mRNAs on day 1 in all three cohorts were *DOCK8-AS1* (1,123 correlated mRNAs), *LEF1-AS1* (1,243 correlated mRNAs), and FAM225A (1,284 correlated mRNAs) (**Figure 2A**). *DOCK8-AS1* is encoded on chromosome 9 and is located on the opposite strand of the *DOCK8* (Dedicator of Cytokinesis 8) gene promoter, while *LEF1-AS1* is an anti-sense lncRNA located in chromosome 4 opposite to the protein-coding gene *LEF1* (Lymphoid Enhancer-Binding Factor 1). *FAM225A* is transcribed form the sense strand of chromosome 9 and its transcription have not been found in other mammalians.

The mRNAs that positively correlated with *FAM225A* and *DOCK8-AS1* were functionally enriched for interferon response and monocytes, whereas the genes negatively correlated with these lncRNAs were enriched for genes commonly expressed by T cells (**Figure 2B**). Monocytes and interferon/innate response were the main upregulated pathways, whereas T cells were the main downregulated ones after rVSV-ZEBOV vaccination. Conversely, genes negatively correlated with LEF1-AS1 were enriched for interferon signaling and monocytes, and genes positively correlated with LEF1-AS1 were enriched for T cell expression (**Figure 2B**). Among the genes that showed a strong (r >0.7) positive correlation with *FAM225* were *IRF7* (**Figure 2C**), a key mediator of the interferon signaling pathway; *IFIH1* (**Figure 2C**), which encodes the viral sensing protein MDA5; and *USP18* (**Figure 2C****),** a gene whose expression in macrophages has been shown to be essential for the effectiveness of the VSV-EBOV vaccine Friedrich Vaccines. *DOCK8-AS1* also presented a highly correlated expression with *IFIH1* and *P2RY13* (**Figure 2C****),** an IFN-stimulated gene that protects against viral infections in mice (18). Both *FAM225A* and *DOCK8-AS1* were negatively correlated with *ZAP70* (**Figure 2C**), which encodes a membrane protein that participates in T-cell receptor (TCR)-mediated antigen activation. LEF1-AS1, on the other hand, had a strong negative correlation with *FCN1* (**Figure 2C**), a gene encoding a collagen-like protein expressed in leukocytes, and *FCER1G* (**Figure 2C**), which encodes an Fc receptor of IgE.

### Vaccine-induced lncRNAs show correlated expression with IgG titers on several days after vaccination

Next, using the VSV-EBOVAC and VSV-EBOPLUS cohorts, we performed a meta-analysis of the correlation between the expression of the 11 VSV-ZEBOV-perturbed lncRNAs and circulating IgG titers at several time points after vaccination. This correlation analysis was performed to identify the potential regulatory roles of lncRNAs in shaping the humoral immune response elicited by the rVSV-ZEBOV vaccine. lncRNAs that exhibit significant correlations with IgG titers across multiple time points may contribute to B cell activation, differentiation, or antibody class switching, which are essential for the vaccine response. Importantly, lncRNA expression was only measured in the early days post-vaccination, whereas IgG titers were measured longitudinally; therefore, the reported coefficients reflect how early lncRNA induction predicts later antibody levels, rather than a correlation between contemporaneous expression and IgG levels at each time point. Patient-level IgG titer data were not available for the VEBCON cohort.

**Figure 3:**
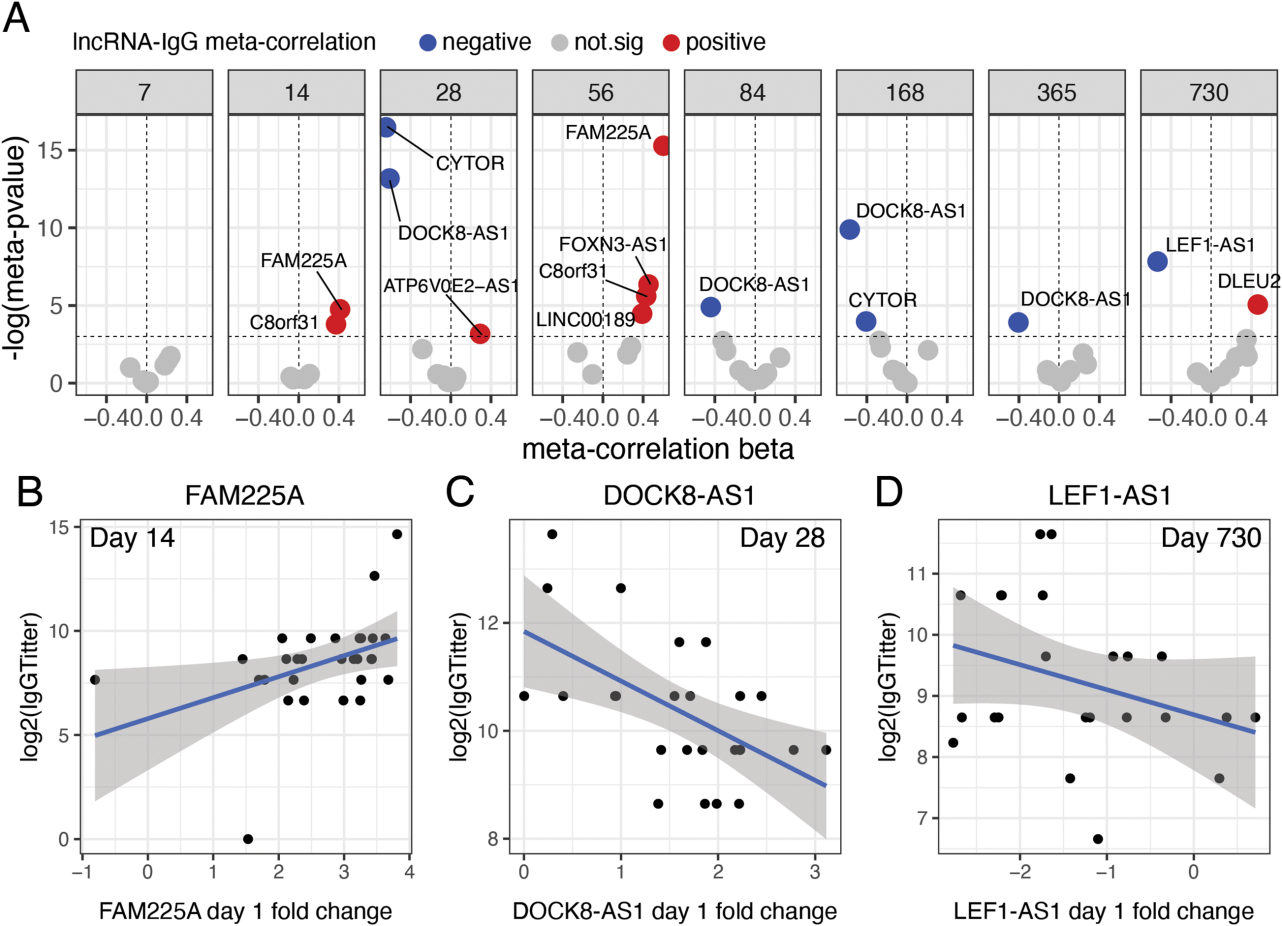
Correlation of lncRNAs with Immunoglobulin G (IgG) titer in volunteers after receiving rVSVΔG-ZEBOV-GP vaccination. **(A)** Significant meta-analysis correlations between day 1 vaccine-induced lncRNAs and IgG titers from 7 days to 2 years after vaccination. Meta-correlation p-values <0.05 were considered significant. Individualized patient baseline normalized (log_2_-scaled D1-D0) expression of *FAM225A*; **(B)** *DOCK8-AS1*; **(C)** and *LEF1-AS1*; and **(D)** versus day 14, 28, and 730 IgG titers (log_2_ scale).

*FAM225A* expression on day 1 after vaccination showed a positive correlation with antibody titers 14 (**Figures 3A, B**) and 56 days post-vaccination (**Figure 3A**). Consistent with its transient induction pattern (**Figure 1C**; **Supplementary Figure S2**), day-1 DOCK8-AS1 levels were negatively correlated with IgG titers on days 28 (**Figure 3C**), 84, 168, and 365 (**Figure 3A**), even though the DOCK8-AS1 expression itself returned to baseline or below at later time points. *LEF1-AS1* expression was negatively correlated with IgG titers only on day 360 (1 year) (**Figures 3A, D**). *CYTOR* presented a negative correlation with antibody levels on days 28 and 168 (6 months) (**Figure 3A**), and *C8orf31* was positively correlated with IgG titers on days 14 and 56 (**Figure 3A**). IgG titers over time for lncRNAs *DOCK8-AS1* and *FAM225A* also showed significant and high correlation scores on other days without the meta-correlation (**Supplementary Figure S2**).

### *DOCK-AS1* can act as a promoter-antisense (PAS) RNA of the mRNA *DOCK8*

**Figure 4:**
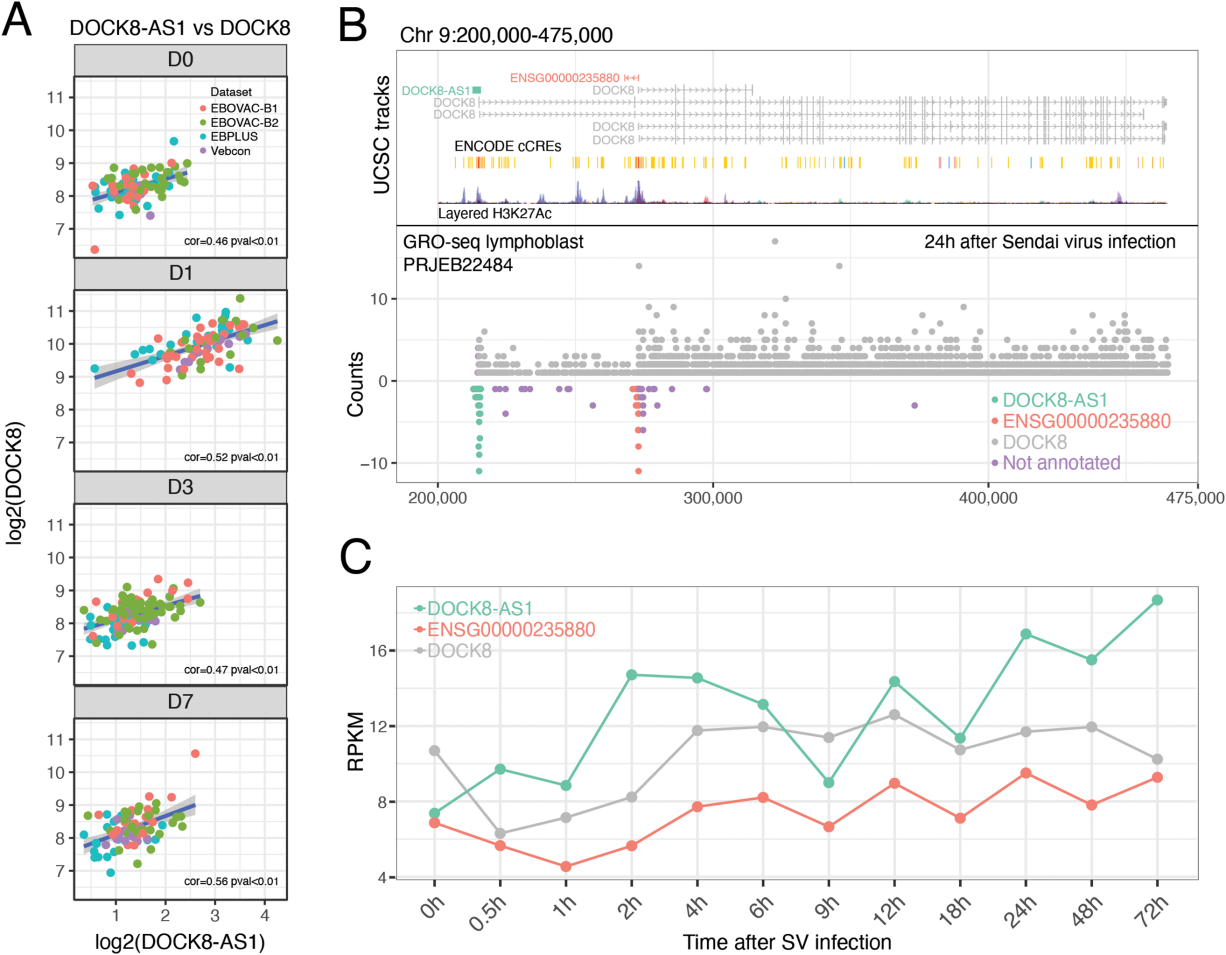
DOCK-AS1 is a putative promoter-antisense (PAS) RNA of the protein-coding gene DOCK8. **(A)** *DOCK8-AS1* expression was positively correlated with *DOCK8* expression in all cohorts and days after rVSVΔG-ZEBOV-GP vaccination. **(B)** Global Run-on sequencing (GRO-seq) analysis of lymphoblastoid cells infected with Sendai virus for 24 h shows concomitant peaks of expression of *DOCK8-AS1* (negative strand) and *DOCK8* (positive strand) around the *DOCK8* gene promoter region. **(C)** *DOCK8* and *DOCK8-AS1* followed a similar pattern of co-expression over time after Sendai virus infection. *ENSG00000235880*, another lncRNA, exhibited a similar pattern.

DOCK8 was one of the genes that presented the highest correlation with the lncRNA *DOCK8-AS1* on all days and in all cohorts (**Figure 4A**), with stronger correlations on days 1 and 7 after vaccination. This correlation was detected even on day 0, before vaccination (**Figure 4A**), demonstrating that *DOCK8-AS1* and *DOCK8* likely have an intrinsically associated pattern of expression even in the absence of salient stimuli. Recently, evidence from global run-on sequencing (GROseq) and CRISPR assays has demonstrated that the expression and shape of promoter antisense lncRNAs (PAS RNAs) are essential for the expression of their neighboring sense-encoded genes (19). Therefore, we hypothesized that *DOCK8-AS1* might play the role of a PAS RNA to induce the expression of *DOCK8* in vaccinated volunteers, leading to the correct migration of T_fh_ cells in response to vaccination. We analyzed a publicly available GROseq dataset of human lymphoblasts infected with Sendai virus probed at several time points from 0 h to 72 h after infection (PRJEB22484) (20) (**Figure 4B**). One day after infection, we detected an accumulation of reads across the entire DOCK8 gene on the sense strand (**Figure 4B**) and a peak of reads mapping to the *DOCK8-AS1* gene on the antisense strand (**Figure 4B**). The GROseq assay allows for the detection of co-occurring *de novo* nuclear RNA expression. This result implies that *DOCK8-AS1* and *DOCK8* are co-expressed at the same time in the nucleus of lymphoblasts infected with the Sendai virus. This pattern was detected at other time points (**Figure 4C**), with evidence that Sendai virus infection is also capable of inducing increased expression of *DOCK8-AS1* and *DOCK8–*24 h after infection (**Figure 4C**), similar to what we observed here for the VSV-ZEBOV Ebola vaccine in the blood.

Curiously, we also detected a peak of antisense reads mapping to the *ENSG00000235880* gene locus in a region inside *DOCK8* with H3K27Ac epigenetic marks correlated with promoter activity (**Figure 4B**). This position within the *DOCK8* gene coincided with the starting position of three shorter alternative first exon isoforms of *DOCK8* (**Figure 4C**). This analysis suggests that both DOCK8-AS1 and ENSG00000235880 are transcribed from bidirectional promoter regions at the canonical and alternative promoters of DOCK8, respectively, in a configuration compatible with promoter-associated antisense (PAS) transcription. Nevertheless, this relationship requires further experimental evidence before concluding that these lncRNAs directly regulate DOCK8 expression.

### LEF1-AS1 may control the expression of mRNA LEF1 and is downregulated upon Ebola vaccination

**Figure 5:**
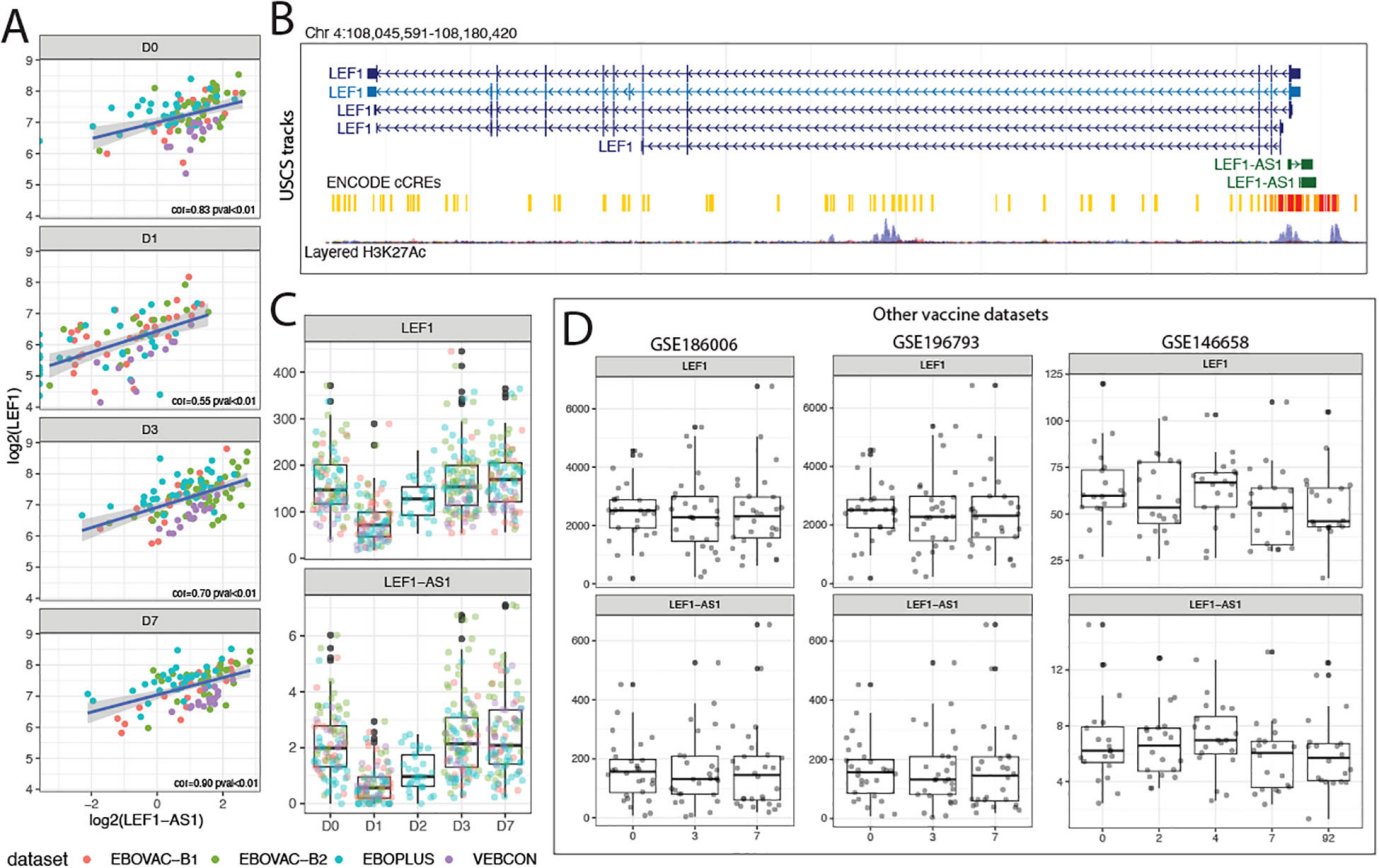
LEF1-AS1 is an antisense lncRNA that prominently regulates LEF1 expression on the first day after vaccination. **(A)** Log2-transformed expression levels of *LEF1-AS1* and *LEF1* across days 0 to 7 post-vaccination in the four cohorts (red for EBOVAC batch 1, green for EBOVAC batch 2, blue for EBOPLUS, and purple for VEBCON). Pearson correlation coefficients and p-values are shown. **(B)** Genomic locations of *LEF1-AS1* and *LEF1* on chromosome 4, showing antisense overlap and proximity to H3K27Ac histone marks and ENCODE candidate cis-regulatory elements (cCREs). **(C)** Counts of *LEF1* and *LEF1-AS1* transcripts after Ebola vaccination. Cohorts are shown in different colors: red for EBOVAC batch 1, green for EBOVAC batch 2, blue for EBOPLUS, and purple for VEBCON. The x-axis shows days post-vaccination, and the y-axis shows the counts of *LEF1* and *LEF1-AS1*. **(D)** Expression of *LEF1-AS1* and *LEF1* in response to other vaccines in independent datasets (GSE186006 and GSE196793 for influenza; GSE146658 for dengue) is shown as boxplots. On the x-axis are days post-vaccination, and on the y-axis are counts of *LEF1* and *LEF1-AS1*.

*LEF1-AS1* is an antisense lncRNA transcribed on chromosome 4 opposite to the protein-coding gene *LEF1* (Lymphoid Enhancer-Binding Factor 1). LEF1 translated protein itself is a positive regulator of lymphocyte development and activation (21), influencing the transcriptional programs that guide immune cell fate. Following rVSV-ZEBOV vaccination, *LEF1-AS1* expression sharply decreased on day 1 and returned to baseline levels on days 2, 3, and 4. *LEF1* exhibited a matching expression profile, and its levels were strongly and positively correlated with those of *LEF1-AS1* (**Figures 5A, B**). *LEF1-AS1* is transcribed from a region upstream of *LEF1*, marked by H3K27Ac histone modifications and other promoter/enhancer marks (**Figures 5B, C**), suggesting a potential promoter-antisense (PAS) RNA regulatory role. Here, we present evidence for *LEF1-AS1* potential ability to regulate the expression of *LEF1*, this regulatory relationship between LEF1-AS1 and LEF1 has been proposed previously in other biological contexts (22). To assess whether this transcriptional regulation pattern was unique to the Ebola vaccine, we compared *LEF1-AS1* dynamics with transcriptomic data from other vaccine trials, including those for influenza and dengue trials. When examining volunteers’ blood expression 3 days after influenza vaccination in two separate cohorts (23) or two days after dengue vaccination (24), the same reduction pattern was not observed (**Figure 5D**). The influenza and dengue vaccines are based on inactivated and live-attenuated viruses, respectively, suggesting that platform differences may underlie the distinct transcriptional responses elicited by the live-replicating viral vector VSVΔG-ZEBOV-GP. Moreover, there was no dataset from day 1 after vaccination transcriptomics available, so a direct comparison with the day 1 potent immunogenic profile of the rVSV-ZEBOV vaccine was not possible. The coordinated downregulation of both *LEF1-AS1* and *LEF1* immediately after Ebola vaccination may reflect a transient suppression of Wnt signaling (22) components to favor early innate immune responses or to modulate antigen-specific T cell activation.

In summary, our multi-cohort analysis of transcriptomic responses to the rVSV-ZEBOV Ebola vaccine revealed a conserved and coordinated induction of long non-coding RNAs (lncRNAs) on day 1 post-vaccination. Among these, 11 lncRNAs showed consistent differential expression across cohorts and were strongly associated with immune gene expression signatures. Several lncRNAs, including *FAM225A*, *DOCK8-AS1*, and *LEF1-AS1* were correlated with key regulators of innate and adaptive immune responses, as well as with the magnitude and durability of vaccine-induced IgG titers. Functional annotation and genomic localization suggest that some of these lncRNAs, particularly *DOCK8-AS1* and *LEF1-AS1*, may act as promoter-antisense RNAs (PAS-RNAs), potentially influencing the expression of adjacent immune genes critical for T cell function and germinal center response.

## Discussion

Vaccination triggers a tightly orchestrated sequence of immune events that begins immediately after antigen exposure. In the case of virus-based vaccines, such as rVSVΔG-ZEBOV-GP, the response is typically characterized by a strong type I interferon (IFN-I) signature, largely driven by potent activation of myeloid cells (6, 25). As a replicating viral vector vaccine, rVSVΔG-ZEBOV-GP may elicit a distinct early innate immune response compared with non-replicating viral vectors or inactivated virus vaccines. This early innate immune response includes TLR and NLR signaling in antigen-presenting cells, resulting in the expression of co-stimulatory molecules and cytokines that guide adaptive responses. Dendritic cells migrate to lymph nodes and present antigens to naïve B and T cells. If the immune environment is sufficiently activated, naïve cells are retained in the lymph nodes to scan for cognate antigens, initiating germinal center (GC) reactions. This process is resource-intensive and tightly regulated, involving T follicular helper (Tfh) cells that support B cell maturation, somatic hypermutation, and class switching from IgM to IgG. The immune setpoint at the time of vaccination can strongly influence the magnitude and quality of subsequent immune responses, with some individuals exhibiting a ‘naturally adjuvanted’ profile that mirrors early adjuvant-induced changes (26). Our group has shown that rVSVΔG-ZEBOV-GP induces a sharp immune activation on day 1 post-vaccination, likely driven by neutrophil clearance, dendritic cell migration, and naïve lymphocyte sequestration in lymph nodes, in contrast to the relatively quiescent immune landscape at day 0, which is necessary to ensure effective priming. (6, 25).

While the dynamics of protein-coding genes in vaccine responses have been extensively characterized, little is known about the role of long noncoding RNAs (lncRNAs). A recent study (27) demonstrated that vaccination elicits robust changes in lncRNA expression, identifying several noncoding transcripts that are tightly regulated post-immunization and play active roles in modulating immune signaling pathways, including antigen presentation and B cell activation, highlighting lncRNAs as integral participants in shaping adaptive responses. In our study, we identified a conserved signature of 11 lncRNAs differentially expressed one day after rVSV-ZEBOV vaccination across three human cohorts, three of which (DOCK8-AS1, FAM225A, and LEF1-AS1) also correlated with antibody titer response. Among the other eight lncRNAs, *CYTOR* (Cytoskeleton Regulator RNA) has been linked to immunotherapeutic responses in cutaneous melanoma (28), regulation of HIV infection (29), and B-cell activation upon Epstein–Barr virus infection (30) *DLEU2* (Deleted in Lymphocytic Leukemia 2) encodes a peptide that facilitates T-regulatory cell induction (31) and is associated with immune cell infiltration in hepatocellular carcinoma (32) and osteosarcoma (33). FOXN3 Antisense RNA 1 (*FOXN3-AS1*) has been implicated in modulating tumor immune infiltration (34) and responses to immunotherapeutic treatment in acute myeloid leukemia (35), while Long Intergenic Non-Protein Coding RNA 189 (LINC00189) shows a correlated expression with CXCL2 in childhood obesity (36), and Long Intergenic Non-Protein Coding RNA 487 (*LINC00487I*) is associated with immune responses in B-cell lymphoma (11). The remaining lncRNAs, *C8orf31*, also known as LYS6-AS1, LY6S Antisense RNA 1) (37), *SERPINB9P1* (Serpin Family B Member 9 Pseudogene 1) (38, 39), and *ATP6V0E2-AS1* (ATP6V0E2 Antisense RNA 1) (40), have been reported in contexts such as cancer, stroke, and other pathological processes but lack direct immune-related characterization.

*DOCK8-AS1* has emerged as a potential promoter-antisense RNA (PAS-RNA) that regulates the expression of *DOCK8*, a gene essential for proper Tfh localization and B cell help in germinal centers (41). Because the Sendai virus GRO-seq data represent a single time-course experiment and GRO-seq measures nascent transcription, our observations at the DOCK8/DOCK8-AS1 locus should be interpreted as qualitative evidence of potential co-activation within a bidirectional promoter rather than proof of direct regulatory causality. *DOCK8* is an essential gene for correct Tfh cell positioning in the germinal center of lymph nodes in response to immune stimuli (42). Mutations in DOCK8 are related to severe immunodeficiency syndrome characterized by hyper-IgE production and recurrent viral and bacterial infections (43), as well as low antibody production and B cell maturation deficiency (44). We observed strong co-expression between *DOCK8-AS1* and *DOCK8* in both vaccinated volunteers and GRO-seq datasets of virus-infected lymphoblasts. Given that *DOCK8* deficiency is associated with impaired antibody class switching and susceptibility to infection (45), the correlation between *DOCK8-AS1* levels and reduced IgG titers at multiple time points suggests a complex, possibly biphasic role in regulating humoral immunity.

While *LEF1-AS1* overexpression in patient blood has been implicated in cancer (46) and COVID-19 pathogenesis (47), it often occurs through a competing endogenous RNA (ceRNA) mechanism. It is known to sponge miRNAs, such as miR-489-3p (48), miR-544a (49), and miR-1285-3p (50). *LEF1-AS1* was consistently downregulated on day 1 post-vaccination, as was its sense gene, *LEF1*. *LEF1* is crucial for lymphocyte development (21), and its regulation by *LEF1-AS1* may represent a transcriptional checkpoint that transiently suppresses developmental programs and enables antigen-driven B cell activation, as found in other leukemic cell lines (51). This downregulation, according to our data, was unique to Ebola vaccination and was not observed in other vaccine datasets (influenza and dengue), which could indicate a potentially vaccine-specific regulatory mechanism. The lncRNA *FAM225A* was positively correlated with IgG titers on days 14 and 56 after vaccination. It also showed a strong correlation with interferon-stimulated genes such as IRF7, IFIH1, and USP18, the latter of which is required for VSV-ZEBOV efficacy in macrophage-mediated responses (52). These data suggest that *FAM225A* may contribute to the initiation of an effective antiviral response and facilitate the transition to durable adaptive immunity via Tfh and GC support.

In addition to their potential cis-like regulatory role, lncRNAs regulate gene expression through various mechanisms. They can recruit chromatin-modifying complexes, such as PRC2, to specific genomic loci, thereby altering local histone marks and transcriptional competence (53). Other lncRNAs modulate transcription factor activity or RNA polymerase II dynamics, influencing transcriptional initiation or elongation (54), whereas some act through RNA–RNA or miRNA interactions, for example, by serving as molecular sponges or guides that shape post-transcriptional control (55). LncRNAs can also function as scaffolds or decoys for protein complexes, integrating multiple regulatory inputs at the RNA level (56). Our correlative transcriptomic analyses cannot distinguish between these mechanisms, and the expression-based regulation we propose for DOCK8-AS1 and LEF1-AS1 should be addressed in future functional studies rather than as a demonstrated mechanism in the context of Ebola vaccination.

We propose a model in which rVSVΔG-ZEBOV vaccination triggers coordinated noncoding RNA regulatory expression that tunes both early innate signaling and subsequent antibody responses. In the first hours to day 1, viral replication and sensing by monocytes and dendritic cells induced a strong IFN-I signature and upregulation of *FAM225A*, together with classical interferon-stimulated genes. We hypothesized that *FAM225A* acts in trans within these myeloid compartments to fine-tune IFN and antigen presentation modules, possibly promoting efficient priming of Tfh and GC B cells, consistent with its positive association with later IgG titers. In parallel, *DOCK8-AS1* is transiently induced together with *DOCK8* in lymphoid cells, potentially acting in cis as a PAS-like RNA that modulates *DOCK8* transcription at its shared promoter. Individual differences in the duration of this early *DOCK8-AS1/DOCK8* module may result in distinct GC trajectories, such that stronger early induction can be associated with reduced long-term IgG levels, as suggested by negative correlations with titers at later time points. Finally, *LEF1-AS1* downregulation at day 1, together with *LEF1*, may transiently relax developmental checkpoints in B and T cells, permitting antigen-driven activation and differentiation, while persistently higher *LEF1-AS1/LEF1* levels in some individuals could limit GC output and contribute to lower year-1 antibody titers. In this model, vaccine-induced lncRNAs form an early “noncoding immune setpoint” that integrates innate sensing, Tfh help, and B-cell fate decisions, ultimately shaping the IgG response.

Collectively, our findings revealed that lncRNAs play a significant and coordinated role in shaping the immune landscape following rVSV-ZEBOV vaccination. They appear to modulate both early innate signaling and the quality of the subsequent antibody response, acting through mechanisms such as the cis-regulation of immune effectors and PAS-RNA-mediated transcriptional control. This is the first study to highlight the role of lncRNAs in the quality and longevity of the humoral response to Ebola vaccination, opening the door for future mechanistic investigations and vaccine-enhancing interventions that target noncoding RNA networks.

## Methods

### Volunteer’s cohorts

Volunteers from the VSV-EBOVAC and VSV-EBOPLUS cohorts participated in clinical trials for the rVSVΔG-ZEBOV-GP Ebola vaccine conducted in Europe and North America. VSV-EBOVAC: phase 1/2, randomized, double-blind, placebo-controlled, dose-finding trial conducted in Geneva, Switzerland (November 2014 to January 2015; NCT02287480). VSV-EBOPLUS: phase 1b, randomized, double-blind, placebo-controlled, dose–response trial in the USA (5 December 2014 to 23 June 2015; NCT02314923). The VEBCON volunteers were participants in the rVSV-ZEBOC clinical trial conducted in Europe: a phase 1, open-label, dose-escalation single-center trial conducted in Hamburg, Germany (November 2014 to December 2014) (4–6).

### Blood samples RNA extraction and RNA sequencing

For the VSV-EBOVAC and VSV-EBOPLUS cohorts, 2.5 mL of venous blood was collected in PAXgene blood RNA tubes (PreAnalytiX, Hombrechtikon, Switzerland) from all participants on days 0, 1, 2, 3, and 7. Comprehensive methods for extraction and sequencing have been previously described (4–6).

### Public data acquisition and experimental methods

For the VEBCON cohort, raw RNA sequencing counts and patient metadata were obtained from the Gene Expression Omnibus (GEO) database (GEO accession: GSE97590) (57). Briefly, RNA was extracted from the blood of participants on days 0, 1, 3, and 7 after vaccination. RNA-seq libraries were generated using the NEBNext Ultra RNA Library Prep Kit for Illumina (NEB) and sequenced on an Illumina HiSeq 2500 (single read, 50-bp run). Reads were aligned to the human reference assembly (GRCh38.p7) using STAR (v2.4.2a) and quantified with “–quantMode GeneCounts.” Gene annotation was obtained from Ensembl (Release 79, http://www.ensembl.org).

### IgG quantification

ELISA assays to detect ZEBOV-GP-specific IgG antibody titers were performed as previously described (58). Briefly, ZEBOV glycoprotein-specific antibodies were quantified using the Filovirus Animal Non-Clinical Group (FANG)-approved ELISA with the homologous Zaire-Kikwit strain glycoprotein, following the USAMRRIID’s standard operating procedure (SOP AP-03-35; USAMRIID ELISA).

### Differential expression analysis

Genes with less than 1 count per million (CPM) in 10 or more samples were removed from the raw count tables prior to the DE analysis. The R package *edgeR* was used to perform DE analysis using the *glmQLFit* function after using the *calcNormFactors* function to normalize counts and the *estimateDisp* function to estimate dispersions (59). DE genes were considered those with an adjusted p-value lower than 0.05 and an absolute log2FoldChange higher than 0.26 (absolute fold change of 20%). DE testing was performed between the samples from volunteers on days 1, 2, 3, and 7 against their baseline levels on day 0 (before vaccination). The R package *biomaRt* was used to convert gene name synonyms from the VSV-EBOVAC and VSV-EBOPLUS DE results to the HUGO Gene Nomenclature Committee (HGNC) format. VEBCON DE genes were converted from Ensembl gene ID to the HGNC format. GENCODE v37 (60) human genome annotation was used to obtain gene type labels for each HGCN ID and to further annotate each gene. Only genes labeled as “lncRNA” in the gene-biotype column, according to the GENCODE annotation, were retained for further DE analyses. The DE analysis results were plotted using the R packages *ggplot2* and tidyomics (61). VSV-EBOVAC batch 1 after filtering presented 24 lncRNAs, VSV-EBOVAC batch 2–36 lncRNAs, VSV-EBOPLUS 58 lncRNAs, and VEBCON 316 lncRNAs.

### Correlation meta-analysis

Baseline normalized (Basenorm) expression values were calculated for each day after vaccination for all volunteers in each cohort. Basenorm consisted of subtracting day 0 expression values from the expression values on each of the subsequent days following the vaccine for each patient. Basenorm expression values of volunteers in each cohort were used to perform pairwise Pearson’s correlation tests (*cor.test* function in R) between selected lncRNAs and all other genes available in the Basenorm expression matrices. Basenorm values were also used to perform pairwise Pearson’s correlation tests (*cor.test* function in R) between selected lncRNAs and IgG titers on all measured days after vaccination for the VSV-EBOVAC and VSV-EBOPLUS cohorts. The R package *metafor* (62) was used to perform a random-effects model meta-analysis of the lncRNA-gene and lncRNA-IgG correlation p-values and correlation coefficients across all cohorts. Correlation pairs between lncRNAs and genes with a meta-adjusted p-value <0.05 were considered significant. Correlation pairs between lncRNAs and IgG titers with a meta-p value lower than 0.05 were considered significant.

### Functional enrichment analysis

Genes with a significant meta-p value correlation were used to perform overrepresentation analysis (ORA) using the Reactome pathways (63) and Blood Transcription Modules (BTMs) (64) databases as references. For the correlation ORA, the genes were split into positively or negatively correlated genes for each lncRNA according to the meta-correlation coefficient. The targets of miRNAs were submitted to separate ORAs for each miRNA. The function *fora* of the R package *fgsea* was used to perform ORA. All genes in the filtered expression tables for each cohort were used as the background universe for ORA. Significantly enriched pathways were those with an adjusted P value <0.05. All correlation and enrichment analysis results were plotted using the R packages *ggplot2* and *pheatmap*.

### Global run-on sequencing analysis

Raw GRO-seq sequences were obtained from the Sequence Read Archive (SRA) under project accession PRJEB22484 (experiment ERX2171558/ERP104165). This experiment has not been linked to any article in SRA before, and the data were reanalyzed as described by Nagari et al. (65). In summary, raw GRO-seq reads were trimmed to remove adapters and poly A tails using Cutadapt and aligned to the human genome (GRCh38.p13) using Burrows–Wheeler Aligner (BWA). Sorted.bam files were generated from the BWA output.sam files using Samtools. The.bam files were filtered using Samtools to extract the genomic regions of DOCK8-AS1 and DOCK8, before downstream analysis. The R package *Rsamtools* was used to import filtered.bam files to R, and an in-house script was used to extract and plot the GRO-seq counts in the genomic region of the DOCK8-AS1 and DOCK8 genes. The R package *ggbio* was used to plot the gene transcript structures.

### Systematic search for other vaccination datasets

To assess whether the transcriptional regulation of *LEF1-AS1* and its association with *LEF1* expression were specific to the Ebola vaccine, a systematic search of publicly available transcriptomic datasets from other human vaccination studies was conducted by searching for RNA-seq studies that included peripheral blood samples collected at multiple time points following vaccination with other viral vaccines in the Gene Expression Omnibus (GEO). The selected studies had public gene-level expression matrices, sample metadata, and multiple time points. No studies with a day 1 time point were found. Four relevant datasets were found: GSE146658 (dengue vaccine), GSE274606, GSE196793, and GSE186006 (influenza vaccines). GSE274606 had no *LEF1-AS1* expression, so it was excluded. Metadata containing vaccine time points or sample identifiers were integrated into the expression tables. All visualizations were generated using the ggplot2 and tidyomics packages (61).

## Data Availability

The original contributions presented in the study are included in the article/**Supplementary Material**. Further inquiries can be directed to the corresponding authors.

## Author’s note

The Ebola virus remains a significant global health threat because of its high mortality rate and potential for widespread outbreaks, underscoring the urgent need for effective and durable vaccines to control future epidemics. Understanding the transcriptional mechanisms underlying immune responses to vaccination is important for improving vaccine design and efficacy. While protein-coding genes have been extensively studied, the role of long noncoding RNAs (lncRNAs) in vaccine-induced immunity remains unclear. Here, we characterized the dynamics of lncRNA expression following administration of the rVSVDG-ZEBOV-GP Ebola vaccine across multiple human cohorts and identified conserved lncRNA signatures associated with both innate and adaptive immunity.
