# Correction: Long non-coding RNAs in response to Ebola virus vaccine-induced immunity

**DOI:** 10.3389/fimmu.2026.1809363

**Published:** 2026-03-03

**Authors:** Izabela Mamede, Thomaz Luschër-Dias, Isabelle Franco Moscardini, Patrícia Gonzales-Dias, Bárbara Marinho, Fernando Marcon, Thiago Dominguez Crespo Hirata, Michael Eichberg, Donata Medaglini, Ali M. Harandi, Claire-Anne Siegrist, Tom H. M. Ottenhoff, Francesco Santoro, Marylyn M. Addo, André Gonçalves, Daniela M. Ferreira, Rafael Polidoro, Glória R. Franco, Paulo P. Amaral, Helder Nakaya, VSV-EBOPLUS Consortium

**Affiliations:** 1 Department of Biochemistry and Immunology, Institute of Biological Sciences, Universidade Federal de Minas Gerais, Belo Horizonte, Brazil; 2 Department of Clinical and Toxicological Analyses, School of Pharmaceutical Sciences, Universidade de São Paulo, São Paulo, Brazil; 3 Bios-Therapy, Physiological Systems for Health S.p.A., Sansepolcro, Italy; 4 Oxford Vaccine Group, Department of Paediatrics, University of Oxford, Oxford, United Kingdom; 5 Laboratory of Genetics and Molecular Cardiology (LGCM), InCor/Clinical Hospital FMUSP (HCMUSP), São Paulo, Brazil; 6 Centre of Omics Technologies (CTO), School of Pharmaceutical Sciences, University of São Paulo, São Paulo, Brazil; 7 Strategic Alliances at Merck Research Laboratories, Boston, MA, United States; 8 Department of Medical Biotechnologies, Laboratory of Molecular Microbiology and Biotechnology, University of Siena, Siena, Italy; 9 Department of Microbiology & Immunology, Institute of Biomedicine, Sahlgrenska Academy, University of Gothenburg, Gothenburg, Sweden; 10 Vaccine Evaluation Center, BC Children’s Hospital Research Institute, University of British Columbia, Vancouver, BC, Canada; 11 Center of Vaccinology, Department of Pathology and Immunology, Faculty of Medicine, University of Geneva, Geneva, Switzerland; 12 Leiden University Center of Infectious Diseases (LUCID), Leiden, Netherlands; 13 Dipartimento di Biotecnologie Mediche, Università di Siena, Siena, Italy; 14 Institute for Infection Research and Vaccine Development (IIRVD), University Medical Centre Hamburg-Eppendorf, Hamburg, Germany; 15 Department for Clinical Immunology of Infectious Diseases, Bernhard Nocht Institute for Tropical Medicine, Hamburg, Germany; 16 German Centre for Infection Research, partner site Hamburg-Lübeck-Borstel-Riems, Hamburg, Germany; 17 Department of Pediatrics, Indiana University School of Medicine, Indianapolis, IN, United States; 18 INSPER Institute of Education and Research, São Paulo, Brazil; 19 Hospital Israelita Albert Einstein, São Paulo, Brazil; 20 Institut Pasteur de São Paulo, São Paulo, Brazil

**Keywords:** Ebola (EBOV), lncRNA, RNA, systems biology, vaccine

The order of authors in the author list of the published paper was erroneously given as:

"Izabela Mamede†, Thomaz Luschër-Dias †,Isabelle Franco Moscardini3, Patrícia Gonzales-Dias,Bárbara Marinho, Fernando Marcon,Thiago Dominguez Crespo Hirata, Michael Eichberg, Donata Medaglini, Ali M. Harandi, Claire-Anne Siegrist, Tom H. M. Ottenhoff, Francesco Santoro, André Gonçalves, Daniela M. Ferreira, Rafael Polidoro, Glória R. Franco, Paulo P. Amaral *, Helder Nakaya*, Marylyn M. Addo and VSV-EBOPLUS Consortium."

The correct author list reads:

"Izabela Mamede†, Thomaz Luschër-Dias†, Isabelle Franco Moscardini, Patrícia Gonzales-Dias, Bárbara Marinho, Fernando Marcon, Thiago Dominguez Crespo Hirata, Michael Eichberg, Donata Medaglini, Ali M. Harandi, Claire-Anne Siegrist,Tom H. M. Ottenhoff, Francesco Santoro, Marylyn M. Addo, André Gonçalves, Daniela M. Ferreira, Rafael Polidoro, Glória R. Franco, Paulo P. Amaral*, Helder Nakaya*, and VSV-EBOPLUS Consortium."

The original version of this article has been updated.

