# Supplementary material for: Long non-coding RNAs in response to Ebola virus vaccine-induced immunity
Source: Front Immunol. 2026 Feb 10;16:1695514. doi: 10.3389/fimmu.2025.1695514 (PMC12929432; doi:10.3389/fimmu.2025.1695514)
Supplement: Supplementary file 1 [file DataSheet1.pdf]

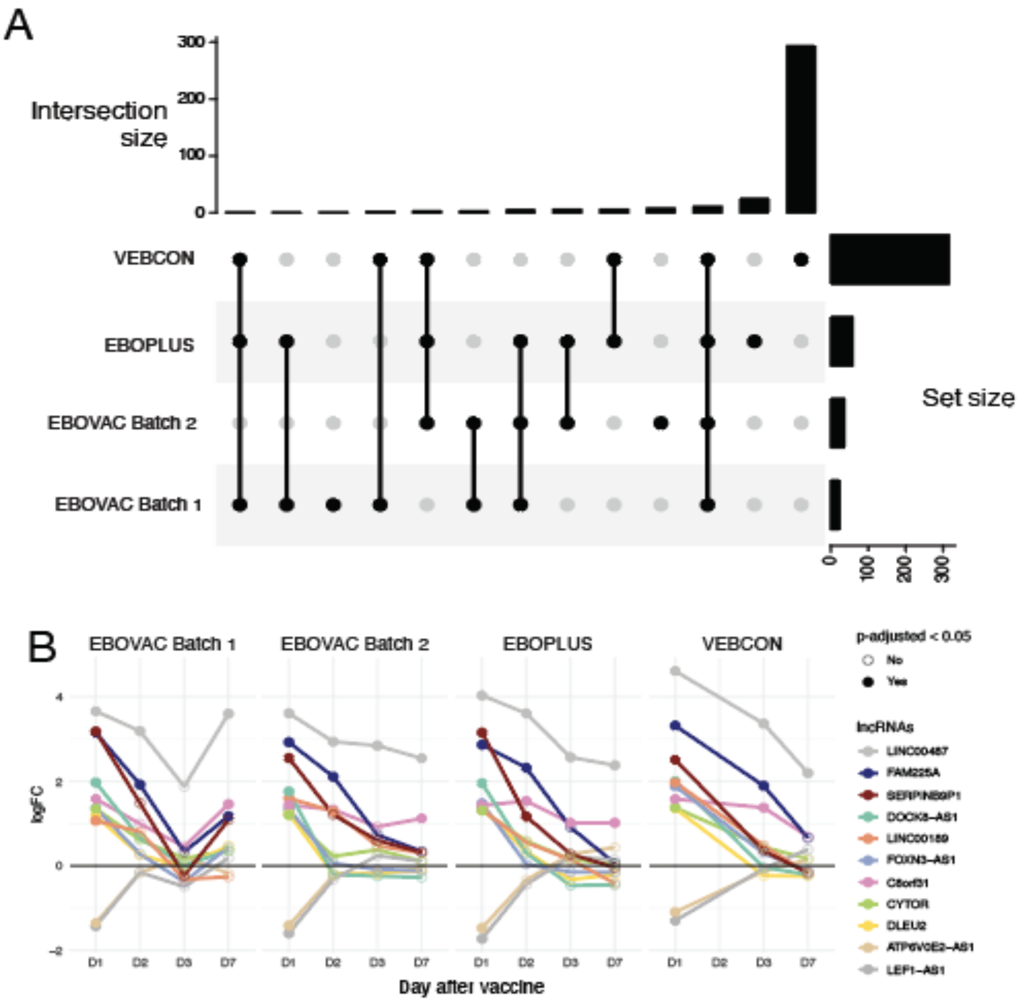

Figure S1: A. Upsetplot showing the intersection of differentially expressed lncRNAs across cohorts. **B**. Line graph of log FoldChange values switch of each selected lncRNA in all the 4 cohorts.

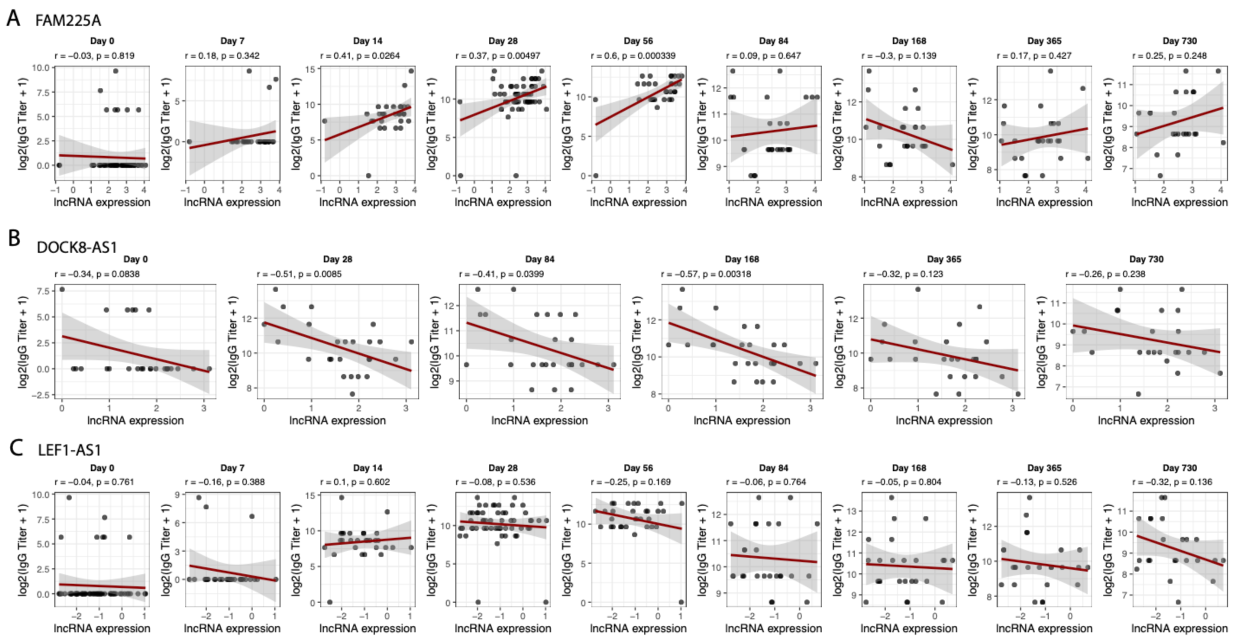

Figure S2: (A–C) Expression of three day 1–induced lncRNAs—*FAM225A* (A), *DOCK8-AS1* (B), and *LEF1-AS1* (C)—correlates with circulating anti-Ebola GP IgG titers across multiple time points after vaccination.

Each subplot shows individualized volunteer expression (log2) versus IgG titer ($\log_2$[IgG + 1]) measured at the indicated day post-vaccination. Pearson correlation coefficients (r) and p-values are shown in each panel.
